# External Validation of the Oakland Score to Assess Safe Hospital Discharge Among Adult Patients With Acute Lower Gastrointestinal Bleeding in the US

**DOI:** 10.1001/jamanetworkopen.2020.9630

**Published:** 2020-07-07

**Authors:** Kathryn Oakland, Sandeepkumar Kothiwale, Tyler Forehand, Edmund Jackson, Cliff Bucknall, Michael S. L. Sey, Siddharth Singh, Vipul Jairath, Jonathan Perlin

**Affiliations:** 1 Department of Digestive Diseases, HCA Healthcare UK, London, United Kingdom; 2 Faculty of Medicine, Imperial College London, London, United Kingdom; 3 Department of Data Science, HCA Healthcare, Nashville, Tennessee; 4 Division of Gastroenterology, Western University, London, Ontario, Canada; 5 Division of Gastroenterology and Biomedical Informatics, University of California, San Diego, San Diego

## Abstract

**Importance:**

Lower gastrointestinal bleeding (LGIB), which manifests as blood in the colon or anorectum, is a common reason for hospitalization. In most patients, LGIB stops spontaneously with no in-hospital intervention. A risk score that could identify patients at low risk of experiencing adverse outcomes could help improve the triage process and allow greater numbers of patients to receive outpatient management of LGIB.

**Objective:**

To externally validate the Oakland Score, which was previously developed using a score threshold of 8 points to identify patients with LGIB who are at low risk of adverse outcomes.

**Design, Setting, and Participants:**

This multicenter prognostic study was conducted in 140 US hospitals in the Hospital Corporation of America network. A total of 46 179 adult patients (aged ≥16 years) admitted to the hospital with a primary diagnosis of LGIB between June 1, 2016, and October 15, 2018, were initially identified using diagnostic codes. Of those, 51 patients were excluded because they were more likely to have upper gastrointestinal bleeding, leaving a study population of 46 128 patients with LGIB. For the statistical analysis of the Oakland Score, an additional 8061 patients were excluded because they were missing data on Oakland Score components or clinical outcomes, resulting in 38 067 patients included in the analysis. The study used area under the receiver operating characteristic curves with 95% CIs for external validation of the model. Sensitivity and specificity were calculated for each score threshold (≤8 points, ≤9 points, and ≤10 points). Data were analyzed from October 16, 2018, to September 4, 2019.

**Main Outcomes and Measures:**

Identification of patients who met the criteria for safe discharge from the hospital and comparison of the performance of 2 score thresholds (≤8 points vs ≤10 points). Safe discharge was defined as the absence of blood transfusion, rebleeding, hemostatic intervention, hospital readmission, and death.

**Results:**

Among 46 128 adult patients with LGIB, the mean (SD) age was 70.1 (16.5) years; 23 091 patients (50.1%) were female. Of those, 22 074 patients (47.9%) met the criteria for safe discharge from the hospital. In this group, the mean (SD) age was 67.9 (18.1) years, and 11 056 patients (50.1%) were female. In the statistical analysis of the Oakland Score, which included only the 38 067 patients with complete data, the area under the receiver operating characteristic curve for safe discharge was 0.87 (95% CI, 0.87-0.87). An Oakland Score threshold of 8 points or lower identified 3305 patients (8.7%), with a sensitivity and specificity for safe discharge of 98.4% and 16.0%, respectively. Extension of the Oakland Score threshold to 10 points or lower identified 6770 patients (17.8%), with a sensitivity and specificity for safe discharge of 96.0% and 31.9%, respectively.

**Conclusions and Relevance:**

In this study, the Oakland Score consistently identified patients with acute LGIB who were at low risk of experiencing adverse outcomes and whose conditions could safely be managed without hospitalization. The score threshold to identify low-risk patients could be extended from 8 points or lower to 10 points or lower to allow identification of a greater proportion of low-risk patients.

## Key Points

**Question:**

Is the Oakland Score a valid tool for assessing the risk of adverse outcomes among a large population of adult patients with acute lower gastrointestinal bleeding in the United States?

**Findings:**

In this prognostic study of 38 067 adult patients who were hospitalized with acute lower gastrointestinal bleeding, the Oakland Score consistently identified patients who were at low risk of experiencing adverse outcomes. Extension of the Oakland Score threshold from 8 points or lower to 10 points or lower for assessing whether a patient can safely be discharged from the hospital could detect more patients who have a low risk of experiencing adverse outcomes and potentially avoid hospitalization in 17.8% of patients whose conditions could safely be managed on an outpatient basis.

**Meaning:**

The findings of this study suggest that adoption of the Oakland Score into the triage process for patients presenting to hospitals in the US could reduce the rate of hospitalization among patients with acute lower gastrointestinal bleeding.

## Teaser

This prognostic study evaluates the external validity of the Oakland Score for use in assessing the risk of adverse outcomes and the likelihood of safe hospital discharge among a large population of adult patients with acute lower gastrointestinal bleeding in the United States.

## Introduction

Lower gastrointestinal bleeding (LGIB) is a common presentation in the emergency departments of hospitals worldwide. In comparison with upper gastrointestinal bleeding (UGIB), LGIB is likely to have a less severe course. Compared with patients with UGIB, patients with LGIB are less likely to present with hemorrhagic shock or require red blood cell (RBC) transfusions or interventions to treat bleeding, and in-hospital mortality rates among patients with LGIB are lower.^1^ Acute LGIB typically presents with bright red rectal bleeding or blood clots from the rectum,^1^ whereas the presenting features of UGIB include hematemesis, coffee-ground emesis, and melena. Unlike patients with UGIB, for which risk stratification scores, such as the Rockall^2^ and Glasgow-Blatchford^3^ scores, are used, patients with LGIB have no equivalent risk score tool available.

In 2016, the American College of Gastroenterology recommended that risk assessment be performed but did not endorse the use of any single tool.^4^ Since the publication of this guideline, the Oakland Score^5^ was developed within a nationally representative sample of patients in the United Kingdom. Rather than calculating a risk score for death or inpatient intervention, the Oakland Score was designed to identify patients who were at low risk of experiencing adverse outcomes and whose conditions could safely be managed without hospitalization. Despite limited external validation of the Oakland Score,^5,6^ national guidelines in the United Kingdom have recently recommended use of the tool for the triage of patients with acute LGIB.^7^ Therefore, the aim of this study was to externally validate the Oakland Score in a large population of patients with acute LGIB from the United States and compare the performance of the Oakland Score at 2 score thresholds (≤8 points vs ≤10 points).

## Methods

Patients with acute LGIB were identified from 140 hospitals in the Hospital Corporation of America (HCA) network across the United States. The study was approved by the HCA Research Review Council. Written informed consent to use unidentified patient data for clinical improvement purposes was obtained from all participants, as written informed consent is built into the overall informed consent process when a patient agrees to receive treatment or diagnostic testing at any HCA hospital. The study used data that are routinely collected for quality improvement purposes, collected no patient identifiers, and involved no new clinical intervention. This study followed the Transparent Reporting of a Multivariable Prediction Model for Individual Prognosis or Diagnosis (TRIPOD) reporting guideline^8^ for prognostic studies.

Adult patients (aged ≥16 years) who were admitted to the hospital with acute LGIB between June 1, 2016, and October 15, 2018, were identified retrospectively using codes from the *International Classification of Diseases, Tenth Revision, Clinical Modification* (*ICD-10-CM*)^9^ that were consistent with a primary diagnosis of LGIB (eMethods in the Supplement). In 2018, the HCA network of US hospitals comprised 179 general and acute care hospitals in 20 states that delivered care to patients who paid for care directly or through Medicare and Medicaid programs, managed care plans, or private insurance. Clinical data from HCA facilities have been stored in a single data warehouse since 2011. For each patient encounter, these data are automatically collected in real time from electronic health records (EHRs) and consolidated in the data warehouse, from which longitudinal EHRs for each patient can be extracted. These EHRs contain data on current hospitalizations as well as comorbidities reported during previous health care events. Hospitals in the HCA network were eligible to participate in the present study if they had an emergency department, routinely admitted patients with emergency conditions, and had comprehensive EHRs to allow record linkage of clinical outcomes.

Patients provisionally identified as having LGIB who received endoscopic hemostasis during esophagogastoduodenoscopy (using *Current Procedural Terminology* [*CPT*] code 43255) were excluded because they were more likely to have UGIB. Data on demographic characteristics, comorbidities, medications, vital signs, blood test results, treatments, and outcomes were collected for each patient. Heart rate (measured in beats per minute), systolic blood pressure (measured in mm Hg), hemoglobin concentration (measured in g/L), platelet count (measured in 10^9^/L), white blood cell count (measured in 10^9^/L), blood urea nitrogen level (measured in mg/dL), creatinine level (measured in mg/dL), albumin level (measured in g/dL), and international normalized ratio were extracted from the results of each patient’s first recorded set of vital signs and blood tests. Previous hospital admission with LGIB was identified through *ICD-10-CM* codes for LGIB that were recorded at any point in the patient’s longitudinal EHR.

Comorbidities were classified using codes from the *International Classification of Diseases, Ninth Revision, Clinical Modification* (*ICD-9-CM*) that were consistent with cancer and cardiovascular, renal, and liver diseases (eMethods in the Supplement). Patient receipt of oral antiplatelet or anticoagulant medications was identified using the Standard Industrial Classification codes M9L and M9P, and these data were extracted only if receipt of the medications appeared in the patient’s regular medication records. To identify medical procedures received, the following *CPT* codes were used: codes 45378 to 45398 for inpatient colonoscopy, code 36430 for RBC transfusion, code 37242 for mesenteric embolization, and codes specific to small bowel, colon, or rectum resection for abdominal surgery for bleeding (full list of *CPT* codes available in eMethods in the Supplement).

The primary outcome was the composite outcome of safe discharge from the hospital,^5^ with safe discharge defined as the absence of all of the following after hospital presentation: in-hospital rebleeding (defined as a decrease in hematocrit concentrations of 20% or more after 24 hours of clinical stability^10^); RBC transfusion; therapeutic colonoscopy, mesenteric embolization, or laparotomy for bleeding; in-hospital death (all causes); and readmission with subsequent LGIB within 28 days. We were able to identify patients who were readmitted to the index hospital or another hospital in the HCA network using *ICD-10-CM* codes (eMethods in the Supplement) but were not able to detect patients who were readmitted to hospitals outside of the HCA network.

### Oakland Score

The Oakland Score was originally derived from prospective data obtained from 2336 patients with LGIB from 143 hospitals in the United Kingdom in 2015 (eMethods in the Supplement), with the aim of identifying patients at low risk of experiencing adverse outcomes.^5^ The total score contains 7 variables (age, sex, previous hospital admission with LGIB, digital rectal examination results, heart rate, systolic blood pressure, and hemoglobin concentration) and ranges from 0 to 35 points, with higher scores indicating greater risk of experiencing an adverse outcome (Table 1).

**Table 1.** Oakland Score Variables

Variable	Score component value
Age group, y	
≤39	0
40-69	1
≥70	2
Sex	
Female	0
Male	1
Previous hospital admission with LGIB	
No	0
Yes	1
DRE results	
No blood	0
Blood	1
Heart rate, beats/min	
≤69	0
70-89	1
90-109	2
≥110	3
Systolic blood pressure, mm Hg	
50-89	5
90-119	4
120-129	3
130-159	2
≥160	0
Hemoglobin concentration, g/dL	
3.6-6.9	22
7.0-8.9	17
9.0-10.9	13
11.0-12.9	8
13.0-15.9	4
≥16.0	0

Abbreviations: DRE, digital rectal examination; LGIB, lower gastrointestinal bleeding.

SI conversion factor: To convert hemoglobin to grams per liter, multiply by 10.

Data on the Oakland Score variables were extracted from patient EHRs. Digital rectal examination findings were not available, so this variable was omitted from the score calculations. When calculating a total Oakland Score, hemoglobin concentration and systolic blood pressure are assigned the highest point weightings (0-22 points and 0-5 points, respectively). Points ascribed to digital rectal examination results are either 1 point if blood is present or 0 points if blood is absent. The remaining variables were used to calculate an Oakland Score for each patient with LGIB.

### Statistical Analysis

Any patients with missing data on score component variables or clinical outcomes were excluded from the statistical analysis of the Oakland Score. The ability of the Oakland Score to predict in-hospital rebleeding, RBC transfusion, therapeutic intervention to control bleeding, in-hospital mortality, subsequent LGIB within 28 days, and the composite outcome of safe discharge was assessed using area under the receiver operating characteristic (AUROC) curves and 95% CIs.

When assessing the performance of a risk score that is designed to identify low-risk patients, sensitivity is the most important outcome.^11^ When clinicians make decisions about early discharge, it is important that patients at high risk are not misclassified as low risk. Previous studies of patients with UGIB have reported that a sensitivity of 95% or more can be used to identify optimal score thresholds for low-risk patients whose conditions would be potentially suitable for outpatient management.^12^ The sensitivity for safe discharge was calculated for point scores of 8 or lower, 9 or lower, and 10 or lower, and the performance of these score thresholds was compared to identify thresholds that would maintain a sensitivity of 95%. Statistical analysis was performed with Python software (Python Software Foundation) using scikit, SciPy, and NumPy arrays. Data were analyzed from October 16, 2018, to September 4, 2019.

## Results

A total of 46 179 patients admitted to the hospital with a primary diagnosis of LGIB were initially identified. Of those, 51 patients received endoscopic hemostasis at esophagogastoduodenoscopy and were excluded because they were more likely to have UGIB, leaving a study population of 46 128 patients (mean [SD] age, 70.1 [16.5] years; 23 091 women [50.1%]) (eFigure in the Supplement). Of those, 3251 patients (7.0%) were receiving oral anticoagulant medications at the time of hospital admission (Table 2). Overall, 17 896 patients (38.8%) received inpatient colonoscopy, 21 629 patients (68.1%; missing data in 14 387 patients) received RBC transfusion, 3097 patients (6.7%) experienced rebleeding during admission, 79 patients (0.2%) received endoscopic hemostasis during colonoscopy, 15 patients (0.03%) received mesenteric embolization, and 1 patient underwent laparotomy for refractory bleeding. The median length of stay was 3 days (range, 1-175 days), 2048 patients (4.4%) died during hospitalization, and 1166 patients (2.5%) were readmitted with subsequent LGIB. Across the study population, the most common diagnoses were diverticular bleeding (10 657 patients [23.1%]), hemorrhoids (5339 patients [11.6%]), and angiodysplasia (2227 patients [4.8%]). An additional 17 957 patients (38.9%) were classified as having an unspecified gastrointestinal hemorrhage, and 3933 patients (8.5%) were classified as having a hemorrhage of the anus and rectum.

**Table 2.** Demographic Characteristics and Presenting Features of Patients Admitted to Hospital With Acute LGIB

Variable used in development data set	Validation data, No. (%) (N = 46 128)			
	Did not meet criteria for safe discharge (n = 24 054)^a^		Met criteria for safe discharge (n = 22 074)^a^	
	Summary data	Proportion of missing data	Summary data	Proportion of missing data
Age, mean (SD)	72.2 (14.7)	0	67.9 (18.1)	0
Sex				
Male	12 019 (50.0)	0	11 018 (49.9)	0
Female	12 035 (50.0)	0	11 056 (50.1)	0
Previous hospital admission with LGIB	880 (3.7)	NA	282 (1.3)	NA
Comorbidity				
Cardiovascular disease	10 826 (45.0)	NA	6834 (31.0)	NA
Cancer	6056 (25.2)	NA	4046 (18.3)	NA
Liver disease	2487 (10.3)	NA	1636 (7.4)	NA
Renal disease	6860 (28.5)	NA	3341 (15.1)	NA
Test result				
Heart rate, mean (SD), beats/min	84 (17.0)	293 (1.2)	81 (16.2)	363 (1.6)
Systolic blood pressure, mean (SD), mm Hg	128 (25.0)	221 (0.9)	140 (24.8)	357 (1.6)
Hemoglobin concentration, mean (SD), g/dL	85 (2.5)	4550 (18.9)	122 (2.1)	3023 (13.7)
Platelet count, mean (SD), 10^3^/μL	248.5 (111.7)	5143 (21.4)	239.0 (90.2)	3404 (15.4)
WBC count, mean (SD), /μL	9.2 (4.5)	4929 (20.5)	8.8 (3.8)	3152 (14.3)
Urea nitrogen level, mean (SD), mg/dL	34.5 (25.0)	6187 (25.7)	22.4 (15.1)	5564 (25.2)
Creatinine level, mean (SD), mg/dL	1.7 (1.8)	5935 (24.7)	1.3 (1.3)	5312 (24.1)
Medication				
Oral antiplatelet	3005 (12.5)	NA	2058 (9.3)	NA
Oral anticoagulant	1995 (8.3)	NA	1256 (5.7)	NA
INR, median (IQR)	1.6 (1.5)	9496 (39.5)	1.2 (0.7)	9243 (41.2)

Abbreviations: INR, international normalized ratio; IQR, interquartile range; LGIB, lower gastrointestinal bleeding; WBC, white blood cell.

SI conversion factors: To convert hemoglobin to grams per liter, multiply by 10; platelet count to ×10^9^/L, multiply by 1.0; WBC count to ×10^9^/L, multiply by 0.001; urea nitrogen to millimoles per liter, multiply by 0.357; creatinine to millimoles per liter, multiply by 76.25.

^a^ Safe discharge was defined as the absence of all of the following: in-hospital rebleeding, red blood cell transfusion; therapeutic intervention to control bleeding, in-hospital death (all causes); and readmission with subsequent lower gastrointestinal bleeding within 28 days.

Overall, 22 074 (47.9%; 95% CI, 47.4%-48.3%) experienced none of the adverse outcomes specified and could be classified as meeting the criteria for safe discharge. A total of 11 056 patients (50.1%) in this group were female. Compared with patients who did not meet the safe discharge criteria, patients who met the criteria were younger (mean [SD] age, 72.2 [14.7] years vs 67.9 [18.1] years, respectively) with fewer comorbidities (26 229 comorbidities vs 15 857 comorbidities) and fewer patients had previous hospital admissions with LGIB (880 admissions vs 282 admissions), and fewer patients were receiving oral anticoagulant or antiplatelet medications (5000 patients vs 3314 patients) at hospital admission.

**Figure 1.**
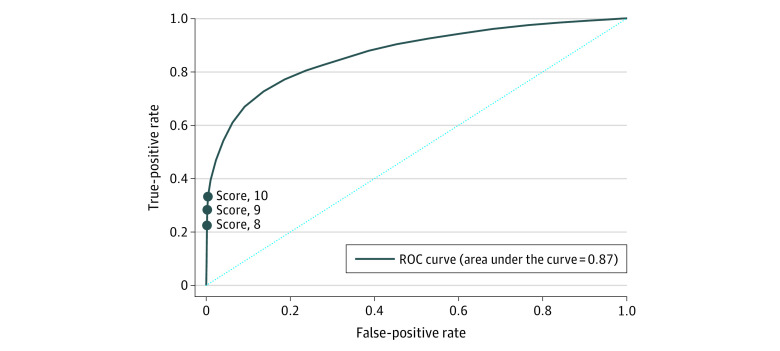
Receiver Operating Characteristic Curve for Safe Discharge ROC indicates receiver operating characteristic.

**Figure 2.**
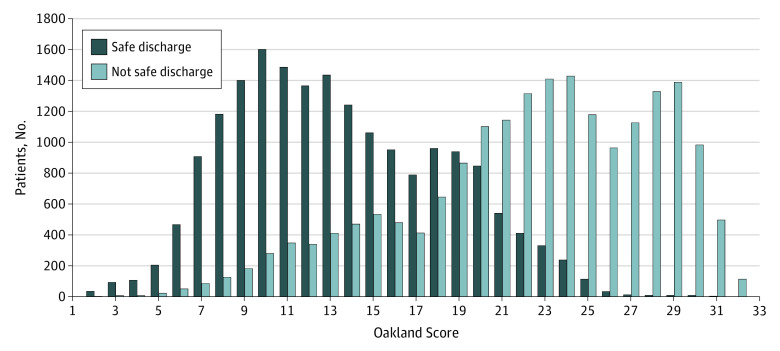
Proportion of Patients Meeting Criteria for Safe Discharge by Total Oakland Score

In total, 38 067 patients (82.5%) had complete data on all components of the Oakland Score and clinical outcomes. The AUROC of the composite outcome of safe discharge was 0.87 (95% CI, 0.87-0.87), suggesting good discriminative performance (Figure 1). In the models predicting adverse outcomes, the AUROCs were as follows: for RBC transfusion, 0.90 (95% CI, 0.90-0.90); for in-hospital rebleeding, 0.46 (95% CI, 0.45-0.47); for death, 0.63 (95% CI, 0.62-0.64); and for hospital readmission with subsequent bleeding, 0.60 (95% CI, 0.59-0.62). Because only 190 patients or fewer (≤0.5%) received therapeutic interventions to control bleeding, AUROCs were not calculated for these outcomes. The median Oakland Score was 18 points (range, 2-33 points) (Figure 2). In total, 3305 of 38 067 patients (8.7%) scored 8 points or lower, with a sensitivity of 98.4% and a specificity of 16.0% for safe discharge (Table 3). A sensitivity of 96.0% for safe discharge was maintained to a score threshold of 10 points or lower, with a specificity of 31.9%. A total of 4888 patients (12.8%) had a score of 9 points or lower, and 6770 patients (17.8%) had a score of 10 points or lower.

**Table 3.** Adverse Outcomes Among Patients With Low-Risk Oakland Scores

Outcome	Oakland Score, No. (%)		
	≤8 Points (n = 3305)	≤9 Points (n = 4888)	≤10 Points (n = 6770)
RBC transfusion	132 (4.0)	236 (4.8)	383 (5.7)
Endoscopic hemostasis	11 (0.3)	16 (0.3)	21 (0.3)
Mesenteric embolization	0	0	0
Surgery	0	0	0
In-hospital rebleeding	153 (4.6)	223 (4.6)	344 (5.1)
In-hospital death	37 (1.1)	60 (1.2)	96 (1.4)
Readmission with subsequent bleeding within 28 d	7 (0.2)	19 (0.4)	39 (0.6)
Any adverse outcome	182 (5.5)	316 (6.5)	507 (7.5)
Safe discharge sensitivity, %	98.4	97.5	96.0
Safe discharge specificity, %	16.0	23.42	31.9

Abbreviation: RBC, red blood cell.

The most common adverse outcomes in patients with Oakland Scores of 10 points or lower were RBC transfusion and in-hospital rebleeding. No patients received mesenteric embolization or surgery, and the percentage of patients who received endoscopic hemostasis was constant between those scoring 8 points or lower (11 of 3305 patients [0.3%]), 9 points or lower (16 of 4888 patients [0.3%]), and 10 points or lower (21 of 6770 patients [0.3%]). Death occurred in 37 of 3305 patients (1.1%) with a score of 8 points or lower, 60 of 4888 patients (1.2%) with a score of 9 points or lower, and 96 of 6770 patients (1.4%) with a score of 10 points or lower (Table 3).

## Discussion

This study of 140 US hospitals in the HCA network found that the Oakland Score could identify patients at low risk of experiencing adverse outcomes who may be safe for hospital discharge. The Oakland Score was able to discriminate patients at low risk of adverse outcomes despite having data on only 6 of the 7 previously validated variables, suggesting that a modification of the total score that does not include the variable of digital rectal examination could be safely used. We also found that the previously recommended threshold of 8 points or lower^7^ to identify low-risk patients could be extended to 10 points or lower while maintaining a sensitivity of 96% for safe discharge.

Consistent with other large observational studies, the most common intervention observed in the present study was RBC transfusion, which was identified in 68.1% of patients. The percentage of deaths (4.4%) is similar to those reported elsewhere (3.4% and 8.8%).^1,13^ Studies from the United States and the United Kingdom indicated that endoscopic hemostasis is performed in only 2.1% to 4.6% of patients with LGIB,^1,14^ but the frequency of less than 1% of patients who received endoscopic hemostasis in the present study is comparatively low. Only 1 *CPT* code is specific for endoscopic hemostasis, but other codes corresponding with nonspecific band ligation or submucosal injection exist. These *CPT* codes may have been used in place of the bleeding-specific code, which could have produced underestimation of the frequency of endoscopic hemostasis in our study. The proportion of patients receiving inpatient colonoscopy was also low, at only 38.8%. Reports on the rates of colonoscopy in a US population of patients with LGIB are rare, as most observational studies use colonoscopy to identify patients. The largest study, performed by Navaneethan et al,^15^ identified hospitalizations of patients with LGIB using *ICD-9-CM* codes. Of the 58 296 patients discharged from the hospital who were identified in that study, only 2270 patients (38.9%) received an inpatient colonoscopy. This percentage is similar to those reported in a smaller US study (34.7%)^16^ and in studies from the United Kingdom^1^ and Australia.^17^

Other scores used to assess risk among patients with LGIB have been developed. The BLEED (ongoing bleeding, low systolic blood pressure, elevated prothrombin time, erratic mental status, and unstable comorbid disease) score^18^ was designed to assess the risk of in-hospital complications, the NOBLADS (nonsteroidal anti-inflammatory drug use, no diarrhea or abdominal tenderness, blood pressure ≤100 mm Hg, antiplatelet drug use, albumin level <3.0 g/dL, disease score ≥2 points, and syncope) score^19^ and the Strate score^10^ were developed to assess the risk of severe bleeding, and the Sengupta score^20^ was designed to assess the 30-day mortality risk.

Risk scores developed for patients with UGIB, such as the AIMS-65 (albumin level <3.0 g/dL, international normalized ratio >1.5, altered mental status, systolic blood pressure ≤90 mm Hg, and age >65 years) score^21^ and the Glasgow-Blatchford score,^3^ have also been evaluated in patients with LGIB. A study by Oakland et al^5^ reported that the AIMS-65 score was the best predictor of death (AUROC, 0.78), the Oakland Score and Glasgow-Blatchford score were the best predictors of rebleeding (AUROC, 0.74), and the Oakland Score was the best predictor of RBC transfusion (AUROC, 0.92). Tapaskar et al^6^ reported that the Oakland Score was the best predictor of severe bleeding (AUROC, 0.74), the Strate score was the best predictor of rebleeding (AUROC, 0.66), and the Glasgow-Blatchford score was the best predictor of RBC transfusion (AUROC, 0.87). Since publication of the Oakland Score, other scores aimed at identifying low-risk patients have been developed. The SHA_2_PE (systolic blood pressure ≥100 mm Hg, hemoglobin level >12 g/dL, hemoglobin level 10.5-12.0 g/dL, no antiplatelet medication, no anticoagulant medication, pulse ≤100 beats/min, and visible bleeding in the emergency department) score^22^ was derived from a study of 580 patients but has not been externally validated.

To assess the generalizability of a prognostic model, the TRIPOD guidelines state that “it is preferable to use a slightly different case-mix in external validation to judge model transportability. Successful external validation studies in diverse settings (with different case-mix) indicate that it is more likely that the model will be generalizable to plausibly related, but untested settings.”^23^^(p214)^ The Oakland Score was originally derived using prospective data from patients in the United Kingdom; however, in the present study, we found that the Oakland Score also has prognostic value in a US population. A strength of the Oakland Score is the simplicity of its components, which include demographic factors, vital signs, and a single blood test, allowing the score to be fully calculated at initial assessment without an observation period or endoscopic findings. A key criticism of the Oakland Score is that because it was designed to be highly sensitive, some patients who might safely be discharged may instead be identified as requiring hospitalization. This possibility is less important than the potential of misclassifying high-risk patients as low risk. The specificities reported in the present article are low (16.0% for an Oakland Score of ≤8 points and 31.9% for an Oakland Score of ≤10 points). These low specificities are consistent with those reported for other risk scores; for example, the specificity of the Glasgow-Blatchford score is 8% to 22% for a score of 0 points and 34% to 39% for a score of 1 point.^11^

Although most patients identified as low risk will not experience adverse outcomes, some patients at each Oakland Score threshold experienced rebleeding, required hospital-based intervention, or died. The same outcomes have been observed in studies of other risk scores that have been adopted into clinical practice.^12,24^ Determining the safe threshold for identification of patients who have a low risk of experiencing adverse outcomes and whose conditions can safely be managed without hospitalization requires balancing the risk of misclassification with the need to identify an adequate real-world population for the score to be clinically useful. In the Oakland Score development study, a score of 8 points or lower was found to be the safe threshold for identifying low-risk patients despite the threshold being applicable to only 8% of patients.^5^ In the present study, an Oakland Score of 8 points or lower also identified only 8.7% of patients. When the score threshold was extended to 10 points or lower, 17.8% of patients were identified as low risk, with a sensitivity for safe discharge of 96%. Nonetheless, because the study population comprised patients admitted to the hospital with LGIB, the proportion of patients scoring 8 points or lower or 10 points or lower would likely be greater if patients who were discharged from the emergency department were included. To determine this proportion, a prospective cohort study is needed, in which all patients presenting to the emergency department are included, regardless of their admission status.

When comparing patient outcomes using a score threshold of 8 points or lower vs 10 points or lower, the number of patients who received hospital-based interventions to treat bleeding remained constant, the percentage of patients who received RBC transfusion increased, and the percentage of patients who died increased. The percentage of deaths at these score thresholds (1.1% at ≤8 points and 1.4% at ≤10 points) is concerning and is similar to the percentage of deaths among low-risk patients reported for the Glasgow-Blatchford score^24^ and other risk scores.^11^ The use of clinician judgment in combination with risk scores is important. The need for RBC transfusion does not necessarily require patient hospitalization because transfusions or iron can be administered in an ambulatory setting. If patients have bled sufficiently to develop symptomatic anemia, they are unlikely to meet the Oakland Score threshold for safe discharge, which allocates at least 13 points to hemoglobin concentrations less than 110 g/L.^5^

Reducing the number of hospitalizations of patients with LGIB has important benefits. Few data are available describing the economic burden of LGIB, but data from a national US database suggested that costs are between $22 142 and $28 749 per hospitalization, or $4492 per bed-day.^15^ Although LGIB is likely to have a more benign course than UGIB, hospitalizations for patients with LGIB are more expensive than those for patients with UGIB, primarily owing to the longer length of stay and higher resource use.^13^ If an Oakland Score threshold of 8 points or lower was used to identify low-risk patients, hospital admission could potentially be avoided in 8.7% of patients. Given that the median length of stay in the present study was 3 days, this reduction in hospital admissions could produce a savings of $44.5 million within the study population alone. If the score threshold was extended to 10 points or lower, this savings could be $91.2 million. A retrospective analysis performed in the United States found that 40% of costs associated with UGIB were incurred after hospital discharge.^25^ Similar findings are likely to apply to LGIB. In addition, because patients with LGIB are likely to be older than 65 years and to have a high comorbidity burden, other reasons for hospital admission may be present.

### Limitations

This study has several limitations. First, patient identification relied on the use of administrative codes; however, previous large studies of patients with LGIB have successfully used *ICD* and *CPT* codes for this purpose.^26,27^ Patients with hospital codes consistent with UGIB were excluded, as were patients who received endotherapy during esophagogastoduodenoscopy. However, the frequency of patients with the discharge code corresponding with an unspecified gastrointestinal hemorrhage suggests that some patients may have had bleeding that originated in the upper rather than the lower gastrointestinal tract, and some may have had small-bowel bleeding. This uncertainty reflects that of clinicians during clinical assessment, in which it is often difficult to distinguish the site of bleeding based on the patient’s medical history and examination alone.

Second, because only 38.8% of patients received inpatient colonoscopy, it is unclear how some of the definitive diagnoses were made. Third, the digital rectal examination variable needed to calculate the Oakland Score was missing; despite this limitation, the score performed well, which provides the option of using this modification in patients who cannot tolerate digital rectal examination, such as those with bleeding from an anal fissure. Fourth, the present study is limited to patents who were hospitalized with LGIB. Fifth, data on RBC transfusion were frequently missing. It is likely that these cases represent an absence of transfusion rather than missing data; however, the missing data may have produced an overestimation of the proportion of patients who received transfusions.

## Conclusions

This large multicenter prognostic study found that the Oakland Score was externally valid for use in assessing the risk of adverse outcomes in patients with LGIB. The Oakland Score threshold of 8 points or lower, which is currently used to identify patients at low risk of experiencing adverse outcomes, could be extended to 10 points or lower to allow identification of a greater proportion of low-risk patients while maintaining sensitivity; however, an increase in adverse events may occur with use of the higher score threshold.

## Notes

Supplement. **eMethods.** Eligible *ICD-9-CM* and *ICD-10-CM* Codes, Laparotomy *CPT* Codes, and Development of the Oakland Score **eFigure.** Flow Chart of Participant Inclusion and Exclusion **eReferences**
