# Supplementary material for: External Validation of the Oakland Score to Assess Safe Hospital Discharge Among Adult Patients With Acute Lower Gastrointestinal Bleeding in the US
Source: JAMA Netw Open. 2020 Jul 7;3(7):e209630. doi: 10.1001/jamanetworkopen.2020.9630 (PMC7341175; doi:10.1001/jamanetworkopen.2020.9630)
Supplement: Supplement. — eMethods. Eligible ICD-9-CM and ICD-10-CM Codes, Laparotomy CPT Codes, and Development of the Oakland Score eFigure. Flow Chart of Participant Inclusion and Exclusion eReferences [file jamanetwopen-3-e209630-s001.pdf]

Supplementary Online Content

Oakland K, Kothiwale S, Forehand T, et al. External validation of the Oakland Score to assess safe hospital discharge among adult patients with acute lower gastrointestinal bleeding in the US. *JAMA Netw Open.* 2020;3(7):e209630. doi:10.1001/jamanetworkopen.2020.9630

eMethods. Eligible *ICD-9-CM* and *ICD-10-CM* Codes, Laparotomy *CPT* Codes, and Development of the Oakland Score

eFigure. Flow Chart of Participant Inclusion and Exclusion

eReferences

This supplementary material has been provided by the authors to give readers additional information about their work.

© 2020 Oakland K et al. *JAMA Network Open*.

eMethods. Eligible *ICD-9-CM* and *ICD-10-CM* Codes, Laparotomy *CPT* Codes, and Development of the Oakland Score

Eligible *ICD-9-CM* and *ICD-10-CM* Codes

Case ascertainment (*ICD-10-CM*):
K64.0,K64.1,K64.2,K64.3,K64.8,K64.0,K64.1,K64.2,K64.3,K64.4,K64.0,K64.1,K64.2,K64.3,K64.8,K57.11,K57.51,K57.01,K57.13,K57.41,K57.53,K57.31,K57.51,K57.91,K57.21,K57.33,K57.41,K57.53,K57.81,K57.93,K62.5,K55.21,K92.1,K92.2

Medical comorbidities:
• Cardiovascular disease (*ICD-9-CM*): 414.06, 414.00, 414.01, 414.2, 414.3, 414.4, 401, 402, 403, 404, 405, 437.2, 398.81, 402.01, 402.11, 402.91, 404.01, 404.30, 404.11, 404.13, 404.91, 404.93, 428

• Cancer (*ICD-9-CM*): 203.xx, 204.xx, 205.xx, 206.xx, 207.xx, 208.xx, 230.xx, 231.xx, 232.xx, 234.xx

• Liver disease (*ICD-9-CM*): 571.0, 571.2, 571.3, 571.4, 571.5, 571.6, 571.8, 571.9, 572.3, 572.8

• Renal disease (*ICD-9-CM*): 585.1, 585.2, 585.3, 585.4, 585.5, 585.6

Laparotomy *CPT* Codes

44140, 44141, 44143, 44144, 44145, 44146, 44150, 44160, 44799, 44204, 44205, 44206, 44207, 44208, 44210, 44238, 44899

Development of the Oakland Score

The Oakland score was developed using multivariable logistic regression modelling of 18 candidate predictor variables including oral antiplatelet, oral anticoagulant, and nonsteroidal anti-inflammatory drugs. The purpose of the Oakland score is to identify patient at low risk of harm; therefore, the primary outcome of interest was safe discharge, a composite outcome defined as the absence of all of the following after presentation: re-bleeding, red blood cell transfusion, endoscopic, radiological, or surgical haemostasis, all cause- in-hospital death, and readmission with further lower gastrointestinal bleeding within 28 days. Seven predictive variables were identified: age, sex, previous hospital admission with LGIB, digital rectal examination findings, heart rate, systolic blood pressure, and hemoglobin. The model was converted to a simplified scoring system using the approach by Sullivan et al.[1] The score was then externally validated in a cohort of 288 patients admitted to 2 UK hospitals between 2007 and 2011 that did not contribute to the derivation cohort.[2]

© 2020 Oakland K et al. *JAMA Network Open*.

eFigure. Flow Chart of Participant Inclusion and Exclusion

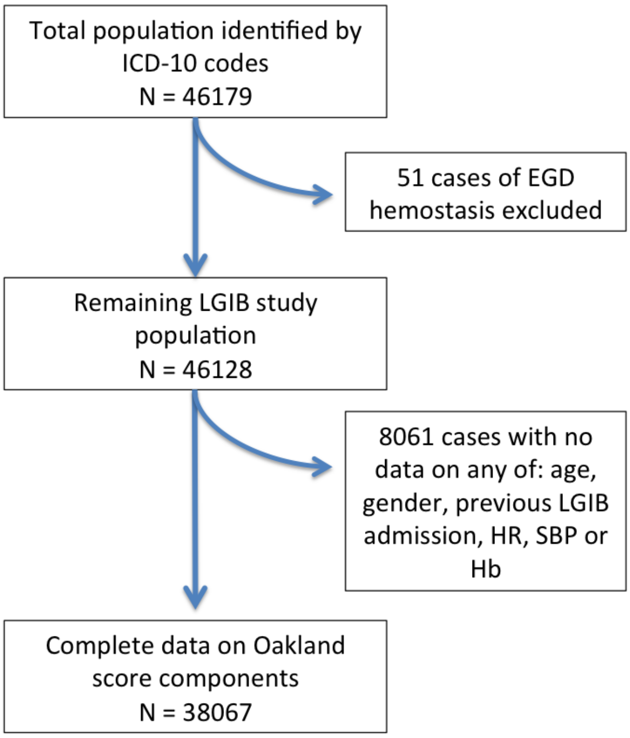

© 2020 Oakland K et al. *JAMA Network Open*.

eReferences

1. Sullivan LM, Massaro JM, D’Agostino RB Sr. Presentation of multivariate data for clinical use: the Framingham study risk score functions. *Stat Med*. 2004;23(10):1631-1660. doi:10.1002/sim.1742

2. Oakland K, Jairath V, Uberoi R, et al. Derivation and validation of a novel risk score for safe discharge after acute lower gastrointestinal bleeding: a modelling study. *Lancet Gastroenterol Hepatol*. 2017;2(9):635-643. doi:10.1016/S2468-1253(17)30150-4

© 2020 Oakland K et al. *JAMA Network Open*.
